# Dysgranular retrosplenial cortex lesions in rats disrupt cross-modal object recognition

**DOI:** 10.1101/lm.032516.113

**Published:** 2014-03

**Authors:** Emma L. Hindley, Andrew J.D. Nelson, John P. Aggleton, Seralynne D. Vann

**Affiliations:** School of Psychology, Cardiff University, Cardiff CF10 3AT, United Kingdom

## Abstract

The retrosplenial cortex supports navigation, with one role thought to be the integration of different spatial cue types. This hypothesis was extended by examining the integration of nonspatial cues. Rats with lesions in either the dysgranular subregion of retrosplenial cortex (area 30) or lesions in both the granular and dysgranular subregions (areas 29 and 30) were tested on cross-modal object recognition (Experiment 1). In these tests, rats used different sensory modalities when exploring and subsequently recognizing the same test objects. The objects were first presented either in the dark, i.e., giving tactile and olfactory cues, or in the light behind a clear Perspex barrier, i.e., giving visual cues. Animals were then tested with either constant combinations of sample and test conditions (light to light, dark to dark), or changed “cross-modal” combinations (light to dark, dark to light). In Experiment 2, visual object recognition was tested without Perspex barriers, but using objects that could not be distinguished in the dark. The dysgranular retrosplenial cortex lesions selectively impaired cross-modal recognition when cue conditions switched from dark to light between initial sampling and subsequent object recognition, but no impairment was seen when the cue conditions remained constant, whether dark or light. The combined (areas 29 and 30) lesioned rats also failed the dark to light cross-modal problem but this impairment was less selective. The present findings suggest a role for the dysgranular retrosplenial cortex in mediating the integration of information across multiple cue types, a role that potentially applies to both spatial and nonspatial domains.

Research into the functions of the retrosplenial cortex has often considered its potential roles in spatial memory (Vann et al. 2009), reflecting its dense interconnections with the hippocampus and anterior thalamic nuclei (Van Groen and Wyss 1990, 1992, 2003). However, other connections of the rodent retrosplenial cortex suggest a broader role in multimodal processing. For example, the retrosplenial cortex receives visual information directly from the geniculostriate and tecto-cortical visual systems (Van Groen and Wyss 1992; Wyss and Van Groen 1992). The retrosplenial cortex also has reciprocal connections with parietal and parahippocampal cortices that may, respectively, provide somatosensory and olfactory information (Insausti et al. 1997; Aggleton 2010). The former inputs are of additional interest given recent evidence of the importance of the parietal cortex for tactile to visual cross-modal transfer in the rat (Winters and Reid 2010). Taking these results together, the retrosplenial cortex becomes a plausible candidate for the integration of different classes of sensory information to help form multisensory representations.

The retrosplenial cortex is divided into two major subregions, granular (area 29) and dysgranular (area 30), which differ in their connectivity and cellular morphology. In the rat, the dysgranular area may be of particular importance for the integration of visual information given its many connections with both cortical and subcortical areas strongly linked to visual processing, e.g., area 17 and the lateral dorsal thalamic nucleus (Vogt and Miller 1983; Van Groen and Wyss 1992). This view is supported by the finding that immediate-early gene expression increases selectively in the dysgranular retrosplenial cortex, rather than the granular retrosplenial cortex, following spatial memory tasks performed in the light, compared with the same tasks performed in the dark (Pothuizen et al. 2009).

Many studies have demonstrated the involvement of the retrosplenial cortex in spatial memory problems, including when the tasks involve cue switching. Examples of the latter include solving a radial-arm maze task in the light and then the dark (Chen et al. 1994), changing from allocentric to directional cues in a T-maze (Pothuizen et al. 2008), and switching from intra-maze to extra-maze cues when performing a radial-arm maze task in the light (Vann and Aggleton 2004, 2005; Pothuizen et al. 2008). It has been argued that the retrosplenial cortex has a “translational” function in transforming spatial codes, e.g., transforming allocentric representations into egocentric ones and vice versa (Burgess et al. 2001; Byrne et al. 2007; Vann et al. 2009). This translation function has, however, largely been applied to spatial processing. The present study used tests of object recognition to explore stimulus translation across a broader domain.

In the standard spontaneous object recognition task (Ennaceur and Delacour 1988) rats are first presented with two identical objects and then, after a retention interval, allowed to explore the now familiar object and a novel, alternative object. Rats preferentially explore the novel object. Previous studies have shown that rats with retrosplenial cortex lesions are unimpaired on standard object recognition tasks (Ennaceur et al. 1997; Vann and Aggleton 2002; Parron and Save 2004). This same task has, however, been modified to examine cross-modal object recognition (Winters and Reid 2010). In this task variant, the types of sensory cue available to the animal at the sample and test phases are switched, e.g., forcing the rats to rely on tactile cues in the sample phase but on visual cues in the test phase. To ensure tactile processing, rats were exposed to objects in the dark, while the subsequent visual discriminations were enforced by placing a clear Perspex barrier between the rat and the test objects in the light (Winters and Reid 2010). Using these same methods (Fig. 1), the present experiment determined whether retrosplenial lesions impair performance when rats are forced to use cross-modal strategies to solve recognition problems. Complementary experiments then examined object recognition memory when rats could only use visual cues or tactile cues throughout the object recognition test. Rats with retrosplenial lesions of both areas 29 and 30, as well as rats with lesions targeted at area 30 alone, were examined. Area 30 (dysgranular) was selected as it is the dominant recipient of visual information within the retrosplenial cortex.

**Figure 1.**
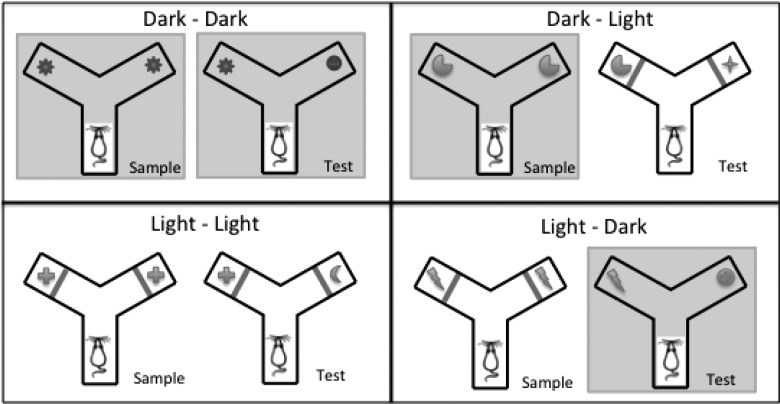
Schematic diagram showing the four different trial types in the cross-modal object recognition task. *Left* images in each pair show the sample phase, while *right* images show the test phase. The different symbols represent different objects. In any light-phase condition, lights were turned on and barriers were placed between the rat and the object to prevent tactile exploration; during dark phases these barriers were removed but the lights were turned off. Light–light and dark–dark trials do not require a cross-modal switch, while dark–light and light–dark trials do.

## Results

### Histological evaluation of the lesions

**Figure 2.**
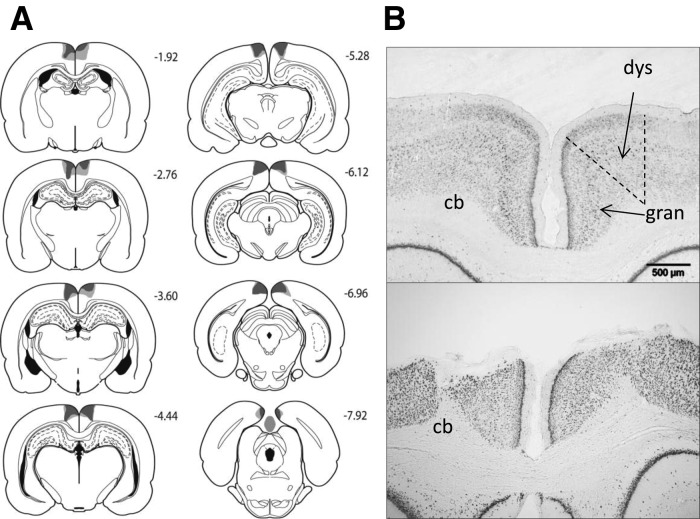
(*A*) Series of coronal sections showing the cases with the largest (light gray) and smallest (dark gray) lesions in the dysgranular retrosplenial (RSdysg) lesion cohort. The numbers correspond to the distance behind bregma in millimeters (Paxinos and Watson 2007). (*B*) Coronal NeuN sections showing the retrosplenial cortex (both hemispheres) from a sham surgery control rat (*top*), and a representative rat from the RSdysg lesion group (*bottom*). (cb) Cingulum bundle; (dys) dysgranular retrosplenial cortex; (gran) granular retrosplenial cortex.

**Figure 3.**
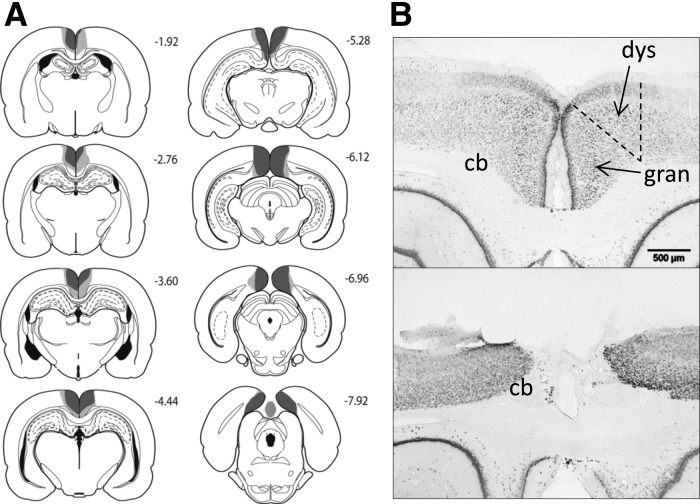
(*A*) Series of coronal sections showing the cases with the largest (light gray) and smallest (dark gray) lesions in the combined dysgranular and granular retrosplenial (RScomb) lesion cohort. The numbers correspond to the distance behind bregma in millimeters (Paxinos and Watson 2007). (*B*) Coronal NeuN sections showing the retrosplenial cortex (both hemispheres) from a sham surgery control rat (*top*), and a representative rat from the RScomb lesion group (*bottom*). (cb) Cingulum bundle; (dys) dysgranular retrosplenial cortex; (gran) granular retrosplenial cortex.

Six rats in the dysgranular retrosplenial lesion group (RSdysg) were excluded as the lesions were either only present in one hemisphere or because there was a high level of sparing of the dsygranular retrosplenial cortex. The final number of animals in the RSdysg group was eight, with 10 in the corresponding sham group (Sham1). In the RSdysg group, none of the remaining animals had damage to the hippocampus or the subiculum. Five of the RSdysg animals had a very limited amount of unilateral damage to the granular retrosplenial cortex (Rgb) (see Figs. 2, 3). The lesions did not extend into any other adjacent cortical areas.

In the combined retrosplenial lesion cohort (RScomb), three rats were excluded due to sparing of the retrosplenial cortex or due to bilateral damage to the hippocampus, leaving 13 rats in the RScomb lesion group and 12 corresponding shams (Sham2). In the RScomb group, extensive cell loss and gliosis was seen throughout the retrosplenial cortex in both the granular and dysgranular subregions. Three animals had restricted damage or gliosis in the most dorsal medial tip of the CA1 subfield of the hippocampus (two unilateral). In the remaining case the bilateral CA1 damage was very restricted. Over all cases, the maximum extent of anterior–posterior hippocampal damage was 600 μm. Seven animals, including the three with CA1 damage, had slight unilateral thinning of the medial blade of the dentate gyrus just caudal to the splenium. Nine animals had partial sparing of Rga, particularly at its caudal limit. Four rats also had some limited sparing of Rgb (see Figs. 2, 3). One rat had slight damage to the anterior cingulate cortex at the junction with retrosplenial cortex, and two showed limited unilateral damage to the secondary motor cortex, lateral to the retrosplenial cortex. A restricted area of gliosis was observed at the junction of the anterior medial and anterior ventral nuclei, as is consistently observed after extensive retrosplenial lesions (Neave et al. 1994; Gonzalez et al. 2003; Vann et al. 2003). No gliosis was seen in this area in the RSdysg cohort.

### Experiment 1—cross-modal object recognition

The cross-modal object recognition task required restricting the cue modalities available in both the sample and the test phases. This was achieved either by running the task in the dark, to remove visual cues, or by inserting a clear Perspex barrier between the animal and the object, to prevent tactile and olfactory exploration. Recognition performance was assessed using the D2 index (Ennaceur and Delacour 1988), which is calculated as a measure of novel object preference, using the formula (time exploring novel object minus time exploring familiar object)/(total object exploration time). This ratio index was preferred to the absolute difference between time spent exploring the novel and familiar objects as the different lighting conditions were associated with changes in overall exploration time.

### Cohort 1—dysgranular retrosplenial cortex lesions

#### Sampling behavior

Across the sample phases there was no difference in the amount of time that the RSdysg and Sham1 animals spent exploring the objects (main effect of lesion on sample exploration time, *F* < 1). However, total exploration times during the sample phase did vary significantly depending on whether visual cues (light) or tactile cues (dark) were available (*F*_(1,16)_ = 94.4, *P* < 0.001) (see Table 1). There was no lesion by lighting type interaction (*F* < 1). The different durations of exploration in the light versus the dark reinforced the decision to focus on the D2 index of recognition for subsequent analyses.

**Table 1.**
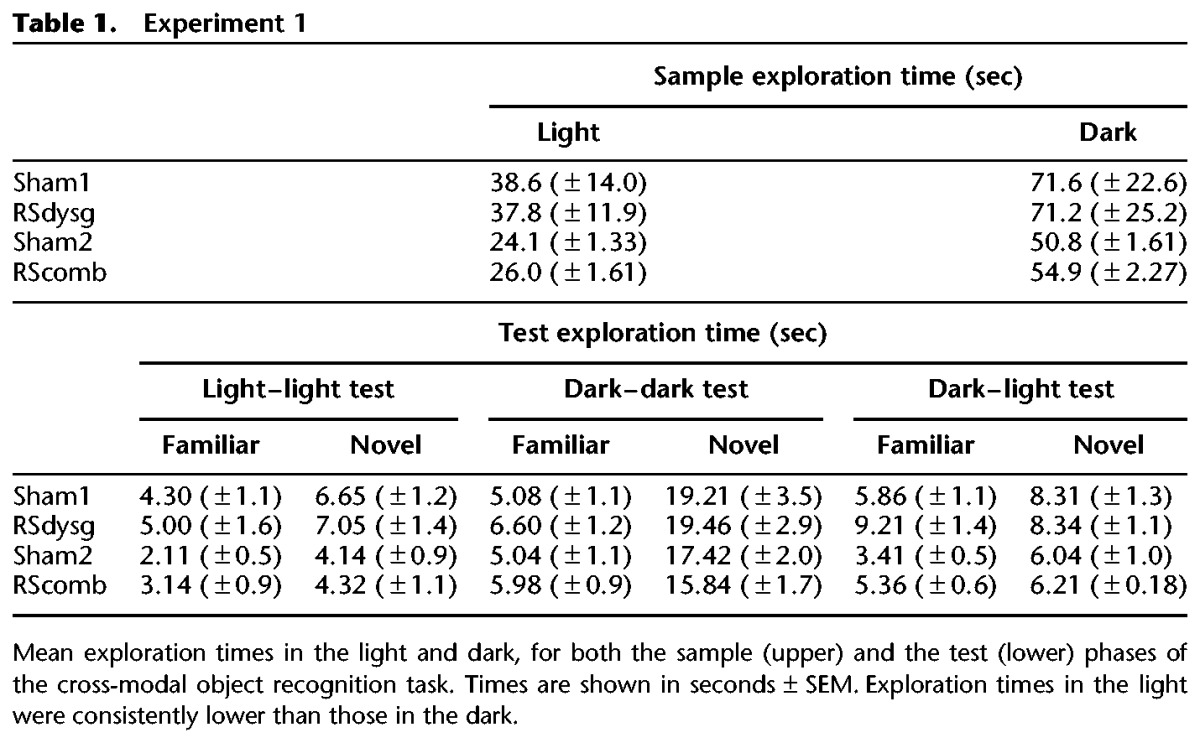
Experiment 1

In the test phase, differing levels of overall exploration were seen in the dark and light tests, with again more exploration in the dark (*F*_(1,16)_ = 230.8, *P* < 0.001). The surgical groups did not, however, differ in their total amounts of exploration time (*F* < 1) (see Table 1). There was no group by lighting condition interaction (*F* < 1).

#### Discrimination behavior

**Figure 4.**
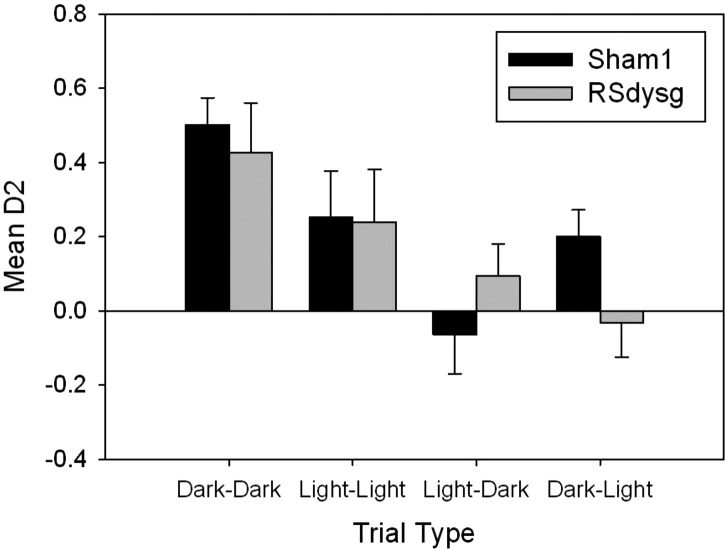
Cohort 1: mean recognition (D2) scores seen at test for the four trial types. Two trial types (dark–dark and light–light) did not require a switch between cue modalities as the same cue types were available at both sample and test. The other two trial types were designed to force the rats to switch between different cue modalities between the sample and the test phases (light–dark and dark–light). Both the Sham 1 and RSdysg groups performed above chance levels on the light–light and dark–dark trials, but only the Sham1 animals were above chance for the dark–light switch. Error bars, SEM.

No differences were found in the D2 scores when the same trial type was repeated (all *F* < 1) and so the two sessions of each trial type were combined prior to their comparison with other trial types. An ANOVA based on the D2 scores with factors of modality (two levels, intra- or cross-modal) and test (two levels, dark or light sample phase) revealed a main effect of modality as the rats discriminated the intra-modal recognition trials more readily than the cross-modal trials (*F*_(1,16)_ = 32.9, *P* < 0.001) (Fig. 4). There was also an interaction between modality and test (*F*_(1,16)_ = 6.5, *P* < 0.05), as performance was better on dark–dark than on light–light trials (*F*_(1,16)_ = 9.8, *P* < 0.05). Neither of these trends was affected by lesion group (max *F*_(1,16)_ = 1.6, *P* = 0.2). However, there was a three-way interaction between modality, test, and lesion (*F*_(1,16)_ 4.6, *P* < 0.05). This interaction arose because the RSdysg group performed worse on the dark–light trials relative to shams (*F*_(1,16)_ = 12.8, *P* < 0.01) but there were no statistically reliable differences between the groups on any of the other trial types (all *F*s < 1). There was also no overall effect of lesion (*F*_(1,16)_ = 1.4, *P* = 0.26).

On the dark–light (cross-modal) trials, tactile cues were available during the sample phase while only visual cues were available during the test phase. The group difference on these trials was due to the Sham1 group significantly discriminating the novel object during the recognition phase (*t*_(9)_ = 4.18, *P* < 0.01), while the RSdysg group's performance was at chance (*t* < 1). It was also found that neither group discriminated the novel object in light–dark trials, where the sample object was explored in the light behind a barrier (visual only), and the test session took place in the dark with tactile cues available (Sham1 *t* < 1, RSdysg *t*_(7)_ = 1.34, *P* > 0.05).

In dark–dark trials, where rats had access to tactile and potential odor cues, but not visual cues, both groups of animals showed a significant preference for the novel object (one-sample *t*-test vs. zero; Sham1 *t*_(9)_ = 12.6, *P* < 0.001; RSdysg *t*_(7)_ = 5.48, *P* < 0.001). Similarly, in light–light trials, when only visual cues could be used, both groups spent more time exploring the novel object (Sham1 *t*_(9)_ = 3.14, *P* < 0.01; RSdysg *t*_(7)_ = 2.52, *P* < 0.05).

### Cohort 2—combined granular and dysgranular retrosplenial cortex lesions

The light–dark condition could not be solved by either group in Cohort 1 (Fig. 4) and so this condition was not included in the experiments with Cohort 2. Since only three trial types were examined, the statistical analyses were modified from those used for Cohort 1.

#### Sampling behavior

Across the sample phases there was no difference in the amount of time that the RScomb and Sham2 animals spent exploring the objects (main effect of lesion on sample exploration time, *F*_(1,23)_ = 1.56, *P* > 0.05). However, total exploration times during the sample phase varied significantly depending on whether the trial was in the light or the dark (*F*_(1,23)_ = 236.0, *P* < 0.001) (see Table 1). There was no lesion by lighting-type interaction (*F* < 1). Once again, the different durations of exploration in the light versus the dark reinforced the decision to focus on the D2 index of recognition for subsequent analyses.

Exploration times during the test phase were found to differ significantly between the light and dark tests (*F*_(1,23)_ = 194.4, *P* < 0.001), with exploration levels in the dark higher than in the light (see Table 1). There were, however, no differences in exploration times between the two groups (*F* < 1), nor was there an interaction with lighting condition (*F* < 1).

#### Discrimination behavior

**Figure 5.**
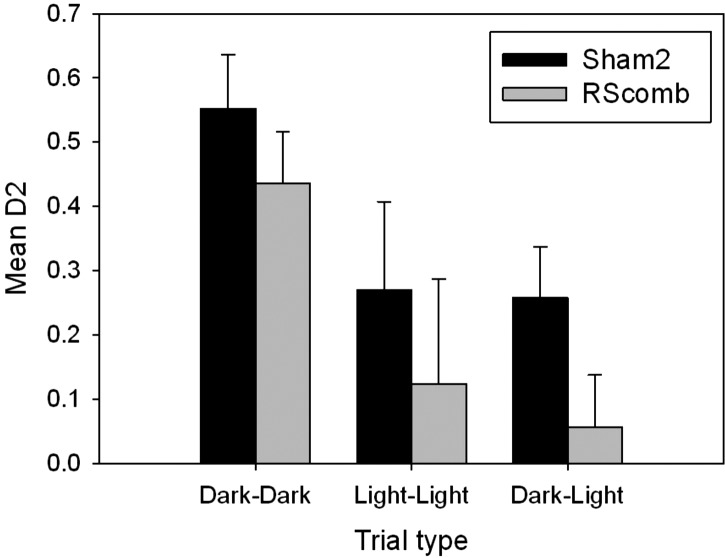
Cohort 2: Mean recognition (D2) scores achieved on dark–dark, light–light, and dark–light trials. While both Sham2 and RScomb rats spent significantly more time with the novel object in dark–dark trials, only the Sham2 rats performed above chance levels in the light–light and dark–light trials on this task. Error bars, SEM.

As with Cohort 1, no differences were found in the D2 scores when the same trial type was repeated (all *F* < 1) and so the two sessions of each trial type were combined. An ANOVA based on the D2 scores revealed an effect of trial type (*F*_(1,23)_ 10.9, *P* < 0.001) as both groups performed best on the dark–dark trials (Fig. 5). There was, however, no interaction between lesion group and trial (*F* < 1), though there was an effect of lesion group (*F*_(1,23)_ = 10.5, *P* < 0.01). This group difference reflected the lower overall D2 scores of the RScomb rats.

On the dark–light (cross-modal) trials, the Sham2 animals scored significantly above chance (*t*_(11)_ = 4.43, *P* < 0.01), i.e., they could recognize stimuli across the modality switch (Fig. 5). In contrast, the RScomb animals failed to show a preference for the novel object (*t*_(12)_ = 1.12, *P* > 0.05) and had lower D2 scores than Sham2 rats (*F*_(1,23)_ = 6.37, *P* < 0.05). Both the Sham2 and RScomb rats could, however, solve the dark–dark trials as they showed a significant preference (D2) for the novel objects (Sham2 *t*_(11)_ = 7.90, *P* < 0.001; RScomb *t*_(12)_ = 7.45, *P* < 0.001) and their D2 scores did not differ (*F*_(1,23)_ = 1.64, *P* > 0.05). While there was no D2 group difference on just the light–light trials (*F*_(1,23)_ = 1.33, *P* > 0.05), only the Sham2 animals showed clear recognition of the novel objects (*t*_(11)_ = 2.87, *P* < 0.05; RScomb animals *t*_(12)_ = 1.44, *P* > 0.05).

### Experiment 2—visual object recognition

One concern with the cross-modal object recognition task is the reduced levels of exploration when an object is placed behind a barrier in the “visual” trials. The aim of Experiment 2 was to identify and test three-dimensional stimuli that would only provide visual cues for a recognition memory test, even though the rats could interact directly with the objects (there was no barrier). Through an initial screening stage (dark–dark trials), objects were selected that the rats could not distinguish by their olfactory or tactile cues. By allowing direct examination in the light (no barrier), object exploration times should increase over those seen in Experiment 1 for the light conditions, so enabling visual-based recognition.

### Cohort 1—dysgranular retrosplenial cortex lesions

**Figure 6.**
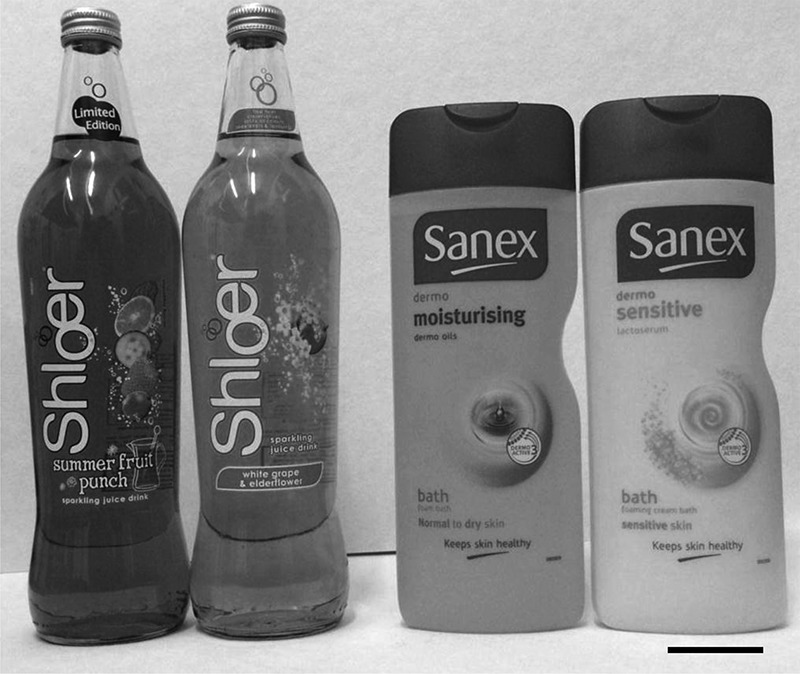
Examples of objects used during the visual object recognition task. Objects were indistinguishable with regard to shape, material, texture, and smell, as determined by the failure of the rats to recognize these objects in the dark. However, these same objects differed in color and pattern, and so could potentially be visually distinguished when subsequently tested in the light. Scale bar, 5 cm.

Those pairs of objects that animals could discriminate in the dark–dark trials were removed from the experiment on the grounds that they had distinctive textures or odors that allowed them to be distinguished nonvisually. Three sets of objects that could not be discriminated in the dark were subsequently used for testing in the light (Fig. 6). The criterion for selection was that the D2 scores of both groups failed to be above chance (one-sample *t*-test, *P* ≥ 0.05, one tailed). For purposes of comparison with the light–light trials, the data from the dark–dark screening trials for those same three pairs of objects are presented and analyzed.

#### Sampling behavior

During the sample phase no differences in exploration time were found between the two lesion groups (*F* < 1), or between the dark and light sample phases (*F*_(1,16)_ = 2.48, *P* > 0.05). There was no sample lighting by lesion interaction (*F* < 1) (see Table 2).

**Table 2.**
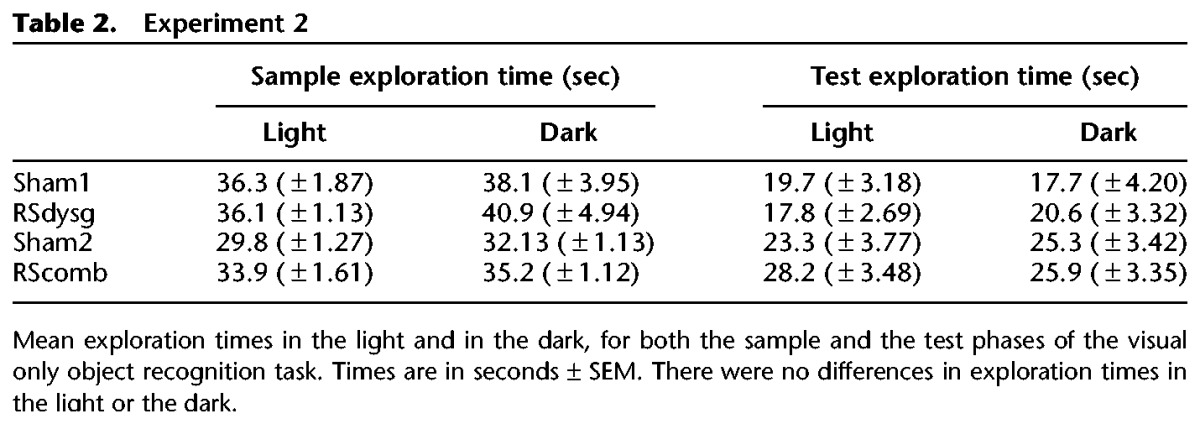
Experiment 2

No group differences were found in exploration time during the choice sessions (*F* < 1), or between the light and the dark (screening phase) trial types (*F*_(1,16)_ = 3.18, *P* > 0.05). As intended, the mean time spent exploring the objects in the light–light test phase (20.11 sec ± 2.76 sec) (see Table 2) proved to be considerably higher than the light–light exploration times in Experiment 1 (13.8 sec ± 2.47 sec).

#### Discrimination behavior

**Figure 7.**
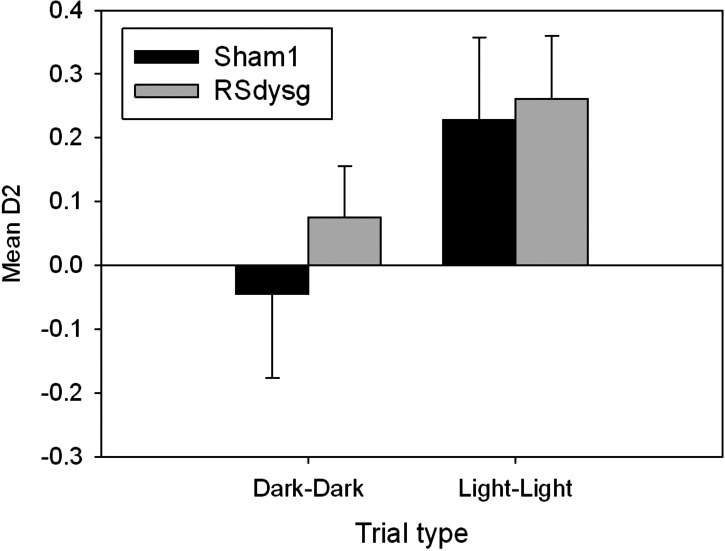
Cohort 1: mean recognition (D2) scores on the dark–dark and light–light sessions of the object recognition task without barriers. Any objects that could be recognized in the dark were removed from the analysis. Consequently, neither the Sham1 nor the RSdysg group was above chance discrimination on the dark–dark trials. On the light–light trials, where the same objects are used as in the dark–dark trials, both groups spent significantly more time with the novel object. Error bars, SEM.

There was no overall difference in recognition (D2) performance between the two groups (*F* < 1) (see Fig. 7, below), though there was a main effect of trial type (*F*_(1,15)_ = 11.5, *P* < 0.01), with performance on the light–light trials superior to the dark–dark trials. This difference is to be expected as all of the objects that were distinguished in the dark were removed from the analysis. As a consequence, neither group performed above chance on the combined dark–dark trials (Sham1 *t* < 1; RSdysg *t*_(7)_ = 1.73, *P* > 0.05). In contrast, both groups of animals showed a significant preference for the novel object in light–light trials (Sham1 *t*_(9)_ = 3.27, *P* < 0.01; RSdysg *t*_(7)_ = 3.38, *P* < 0.05) indicating that they were able to discriminate the objects on the basis of visual cues alone.

### Cohort 2—combined granular and dysgranular retrosplenial cortex lesions

#### Sampling behavior

During the sample phase the lesioned animals tended to explore the objects for longer than the shams (*F*_(1,23)_ = 11.26, *P* < 0.05). However, there was no significant difference between the dark and light sample phases, and no sample lighting by lesion interaction (both *F* < 1) (see Table 2).

During the test phases, the dark–dark and light–light trials did not differ in the amount of time that the rats spent exploring objects in these two conditions (*F* < 1) and no differences were seen between the exploration times of the two groups (*F* < 1) (see Table 2).

#### Discrimination behavior

**Figure 8.**
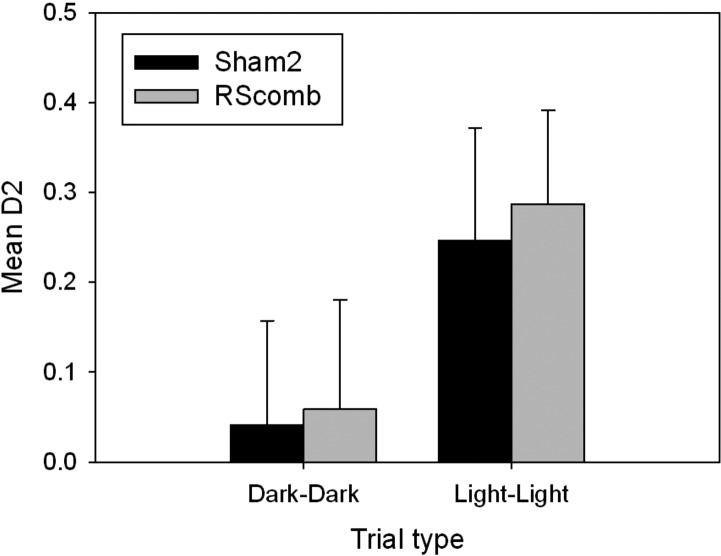
Cohort 2: mean recognition (D2) scores of the Sham2 and RScomb groups on the dark–dark and light–light sessions of the object recognition task without barriers. Any objects that had been recognized in the dark were removed from the analysis. Consequently, neither group was above chance on the dark–dark trials. On the light–light trials, where the same objects are used as in the dark–dark trials, both groups spent significantly more time with the novel object. Error bars, SEM.

As expected, given that any objects that could be recognized in the dark were removed from the analysis, neither group performed above chance level on the dark–dark trials (Sham2 *t* < 1, RScomb *t* < 1). However, both groups had D2 scores significantly above chance on the light–light trials, showing that they were able to distinguish the novel from familiar object when reliant on visual cues (Sham2 *t*_(11)_ = 3.53, *P* < 0.01, RScomb *t*_(12)_ = 3.37, *P* < 0.01) (see Fig. 8). There was no difference between the D2 scores achieved by the Sham2 and RScomb groups on the light–light trials (*F* < 1).

## Discussion

Studies into the functions of the rodent retrosplenial cortex have often focused on its potential role in navigation and spatial memory (Whishaw et al. 2001). While mild deficits are sometimes seen after retrosplenial lesions on standard spatial memory tasks (Vann and Aggleton 2005; Pothuizen et al. 2008; Aggleton 2010), these deficits often become more apparent when rats are forced to switch between different types of spatial information. Examples include moving from using visual to non-visual cues when navigating in the light and in the dark (Cooper and Mizumori 1999), or when switching from local to distal cues (Vann and Aggleton 2004; Pothuizen et al. 2008). From these tasks, however, it is difficult to determine whether the lesion deficit stems from the need to integrate different classes of spatial information or from the related need to switch effectively between these cue types. It is also unclear whether the putative involvement of the retrosplenial cortex in these translational abilities is restricted to spatial information. For these reasons, the present task examined the importance of the retrosplenial cortex for a recognition task that involves integrating and switching between nonspatial cues from different sensory modalities.

The first issue is whether recognition memory within a constant modality is affected by retrosplenial cortex lesions, as any such deficit could confound interpretation of the cross-modal task. Previous studies have found that rats with retrosplenial cortex lesions can often perform at normal levels on standard object recognition memory tasks in the light (Ennaceur et al. 1997; Vann and Aggleton 2002; Parron and Save 2004). In these tasks, rats are typically given three-dimensional objects so that the animal could potentially use visual cues, tactile cues, olfactory cues, or a combination to help recognize the same object when it is presented again. There is, however, a lack of information about the importance of the retrosplenial cortex when performance is limited to just one of these sensory modalities. The present focus on just one modality is supported by studies of object recognition in the dark that have confirmed the ability of rats to use nonvisual cues (Winters and Reid 2010; Albasser et al. 2013) and shown that, unlike object recognition in the light, this form of recognition need not be dependent on the perirhinal cortex (Winters and Reid 2010; Albasser et al. 2013). Rather, it has been found that tactile-based recognition memory is dependent on the posterior parietal cortex (Winters and Reid 2010). Such findings raise the question of whether the retrosplenial cortex might also be involved in non-visual object recognition, despite the lack of any consistent lesion effects when tested in the light.

The present study examined object recognition memory when the animals were restricted from using visual cues, by testing in the dark under infrared illumination. It is known that rats are unable to see in the infrared spectrum (Deegan and Jacobs 1993; Burn 2008), and the results from both Experiments 1 and 2 help to confirm that the rats could not use visual cues in the dark. Thus, in Experiment 1 the rats failed to solve the light–dark cross-modal task, which could have been solved had they been able to use visual cues in the “dark.” Likewise, neither group performed above chance on the selected dark–dark trials in Experiment 2, where visual cues would have aided performance, as demonstrated by their performance with the same objects in the light.

It was found that both rats with dysgranular retrosplenial cortex lesions (RSdysg) and rats with combined lesions of the granular and dysgranular subregions (RScomb) performed as well as their respective sham groups when restricted to the use of tactile or olfactory cues to perform an object recognition task (Experiment 1). That neither lesion impaired recognition in the dark provides a contrast with posterior parietal cortex lesions (Winters and Reid 2010). The RScomb group did, however, show a depression in recognition scores across Experiment 1 that was evident in the light–light trials, where the group failed to perform significantly above chance. This failure suggested a potential, selective problem with visual recognition for the RScomb rats. It was, however, observed that the object exploration times in Experiment 1 were low whenever the object was placed behind a Perspex barrier (for the light trials). This reduction in exploration, which was presumably a consequence of the rat not being able to physically explore the object, would explain the lower D2 scores by all groups on such trials, and may partly explain the seemingly poor RScomb performance. For this reason, a separate visual object recognition task was carried out in Experiment 2, where animals were able to interact directly with the objects. Objects were first screened to select those that could only be distinguished visually, i.e., animals were unable to discriminate the same objects in the dark. When then tested in the light, the rats showed increased exploration times and both the RScomb and RSdysg groups, as well as their respective control groups, were able to discriminate the novel objects (Experiment 2). This result indicates that neither the RScomb nor RSdysg lesions normally affect visual object recognition, consistent with previous findings from standard object recognition testing in the light (Ennaceur et al. 1997; Vann and Aggleton 2002; Parron and Save 2004).

The main goal was to determine the rats’ ability to discriminate novel objects when required to switch cue modalities. The retrosplenial cortex was examined as it is interconnected with sites providing sensory information from multiple modalities, including areas implicated in rodent cross-modal recognition (Winters and Reid 2010; Reid et al. 2012, 2013). A shorter retention interval was used than in previous cross-modal studies (Winters and Reid 2010) in order to avoid floor effects and facilitate comparisons with other studies of object recognition that have also used intervals of 15 min or less (Ennaceur et al. 1997; Vann and Aggleton 2002; Parron and Save 2004); such intervals are sufficient to reveal impairments in object-in-place recognition following retrosplenial cortex lesions (Vann and Aggleton 2002).

The Sham animals performed above chance levels in the cross-modal dark–light trials, consistent with previous studies (Winters and Reid 2010). However, both the RSdysg and RScomb animals failed to solve this trial type, i.e., when there was a switch in cue modalities from tactile to visual between sampling the objects and being tested for recognition. For the RSdysg group this deficit is unlikely to reflect particular problems with either visual or tactile object recognition, as these rats did not differ from their respective sham controls on those tasks. It is notable that posterior parietal cortex lesions also disrupt tactile-visual cross-modal recognition (Winters and Reid 2010) given the connections between the two regions. It was, however, found that parietal lesions also disrupted dark–dark object recognition (Winters and Reid 2010) and so the present findings for the dysgranular retrosplenial cortex provide a more selective deficit of cross-modal transfer. The findings for the combined granular and dysgranular retrosplenial lesions are a little harder to interpret as in Experiment 1 the cross-modal deficit was not selective. In this respect, the results were more like those for parietal cortex lesions (Winters and Reid 2010). Finally, the light–dark trials proved to be particularly difficult for all rats. This result is to be expected as the cross-modal switch followed a sample phase in the light that typically involved significantly less object exploration than found in the dark.

Other relevant information comes from studies of cross-modal matching by monkeys with lesions in the medial temporal lobe (Goulet and Murray 2001) and from fMRI and PET studies of humans performing cross-modal recognition tasks (Banati et al. 2000; Holdstock et al. 2009) (see also Vargha-Khadem et al. 1997). In addition to the perirhinal cortex, which has been implicated in selective aspects of cross-modal recognition in animal studies (Goulet and Murray 2001; Winters and Reid 2010; Albasser et al. 2011), human imaging studies have implicated the anterior cingulate cortex and dorsolateral prefrontal cortex in tactile to visual cross-modal recognition (Banati et al. 2000) along with the insula (Holdstock et al. 2009). It is notable that many of these areas are closely connected with the retrosplenial cortex (Deacon et al. 1983; Kobayashi and Amaral 2003, 2007). These results suggest a network of cingulate and frontal areas that work together to enable cross-modal transfer (Winters and Reid 2010; Reid et al. 2013).

The current results highlight the likely importance of the dysgranular subregion of the retrosplenial cortex for cross-modal recognition. This involvement may reflect the fact that the dysgranular retrosplenial cortex is especially closely connected with brain regions receiving visual information (Van Groen and Wyss 1992) and the way that the present study deliberately isolated demands on vision. While the current findings do not make it possible to determine the involvement of the granular retrosplenial cortex in this task, the results clearly indicate that the rodent retrosplenial cortex has a selective role in integrating or switching between stimuli across modalities, which is not limited to the spatial domain (Byrne et al. 2007). However, it remains difficult to determine whether this cross-modal object recognition deficit stems from an inability to switch between cue types or an inability to associate cue types from different modalities into a single representation, a process that would presumably be required prior to switching. Indeed, a strict division between these two processes may for this reason be too simplistic. However, some preliminary evidence comes from the finding that rats with retrosplenial damage are impaired on acquiring a serial feature negative discrimination task, where rats formed an association between a visual stimulus and a tone (Robinson et al. 2011). The implication is that the retrosplenial cortex might be involved in the initial stimulus–stimulus association process for cross-modal learning. At the same time, retrosplenial cortex lesions do not bring about a general inability to form associations (e.g., Vann and Aggleton 2002; Keene and Bucci 2008; Pothuizen et al. 2008). Such findings highlight the value of examining further the roles of the retrosplenial cortex in stimulus integration and translation, while trying to narrow down the specific nature of its contribution.

## Materials and Methods

### General methods

The study comprised two cohorts of rats. The first cohort consisted of rats with dysgranular retrosplenial lesions and their sham surgical controls, the second cohort consisted of rats with combined granular and dysgranular retrosplenial lesions and their surgical controls. The methods for Cohorts 1 and 2 were almost identical, with a few minor alterations made for Cohort 2 based on the results from Cohort 1.

#### Animals

The two cohorts (dysgranular retrosplenial and granular plus dysgranular) comprised 52 experimentally naive male Lister Hooded rats (Harlan, Bicester, UK). The rats at the time of surgery in the dysgranular cohort weighed from 294 to 314 g. The rats in the combined retrosplenial lesion cohort weighed from 278 to 387 g. The rats were housed in pairs in a temperature-controlled room. Lighting was kept on a 12-h light/dark cycle, light from 8 a.m. to 8 p.m. Water was available ad libitum throughout the experiments. For all behavioral experiments the animals were placed on a food-restricted diet where they were able to gain weight. Their weights did not fall below 85% of their free-feeding weights. All experiments were carried out in accordance with UK Animals (Scientific Procedures) Act, 1986 and associated guidelines. Rats were provided with cardboard tubes and wooden chew sticks in their home cages. Rats in the dysgranular cohort received either a bilateral excitotoxic lesion within area 30 of the retrosplenial cortex (RSdysg *n* = 14) or a sham lesion (Sham1, *n* = 10). Animals in the “combined” lesion cohort received either a bilateral excitotoxic lesion of both areas 29 and 30 (RScomb, *n* = 16) or a sham lesion (Sham2, *n* = 12).

#### Surgical procedures

Rats were deeply anesthetized with an intraperitoneal (i.p.) injection of sodium pentobarbital (60 mg/kg pentobarbital sodium salt; Sigma-Aldrich). All subjects were given a subcutaneous injection of 0.06 mL Metacam (Boehringer Ingelheim) to reduce post-operative pain, as well as 0.1 mL Millophylline (Arnolds Veterinary Products, Ltd.) to regulate breathing. The scalp was shaved and the animal was placed in a stereotaxic frame (David Kopf Instruments) with the nose bar set at +5.0. The skull was exposed and a bilateral craniotomy extending from bregma to lambda was made in the skull using a dental drill. The more posterior areas of the retrosplenial cortex were revealed by drilling away two short strips of skull from the opened area, leaving a strip of bone ∼2 mm wide over the central sinus as protection.

Lesions were made by injecting 0.09 M *N*-methyl-D-aspartate (NMDA; Sigma) dissolved in phosphate buffer (pH 7.2), into 14 injection sites at a rate of 0.05 µL per minute using a 1-µL Hamilton syringe (gauge 25 sec; Bonaduz). The stereotaxic coordinates of the lesion placements are stated relative to bregma in the anterior-posterior (AP) axis, and relative to the central sinus in the lateral-medial (LM) axis. Dorsal-ventral (DV) coordinates were taken relative to the surface of the cortex, using the eye of the needle.

Coordinates for the dysgranular lesion group were: AP-1.6, LM ± 0.4, DV-1.0; AP-2.8, LM ± 0.5, DV-1.1; AP-4.0, LM ± 0.5, DV-1.1; AP-5.3, LM ± 0.5, DV-2.4; AP-5.3, LM ± 0.9, DV-1.4; AP-6.6, LM ± 0.9, DV-1.8; AP-7.5, LM ± 1.0, DV-1.1. At each site 0.25 µL NMDA was injected, apart from for the most caudal pair of injections, where the injections were 0.1 µL NMDA. The coordinates for the combined lesion cohort were AP-1.6, LM ± 0.4, DV-1.3; AP-2.8, LM ± 0.5, DV-1.3; AP-4.0, LM ± 0.5, DV-1.3; AP-5.3, LM ± 0.5, DV-2.6; AP-5.3, LM ± 0.9, DV-1.6; AP-6.6, LM ± 1.0, DV-2.0; AP-7.5, LM ± 1.1, DV-1.3. In each of the three most rostral injection sites, 0.25 µL NMDA was injected. In the next three pairs of sites, 0.26 µL NMDA was injected. In the most caudal site only 0.1 µL NMDA was injected.

After each infusion the needle was left in place for 5 min before being slowly withdrawn. If required, animals were given a single 0.05-mL injection of sodium pentobarbital to maintain anesthesia. If further anesthesia was still required <2% inhaled isoflurane was given. Oxygen was provided throughout the surgery. Following surgery, the scalp was sutured and a subcutaneous injection of 5 mL glucose-saline was given to replace lost fluids. Lidocaine (Xylocaine, AstraZeneca) and antibiotic powder (Dalacin C, Pharmacia) were applied topically to the wound and animals were left to recover in a warm quiet area before being returned to their home cage. Sham animals underwent the same procedure, except that the needle was not lowered and injections of neurotoxin were not made. Post-operative care was identical for all groups. All animals recovered well following surgery.

#### Histological procedures

At the completion of all experiments, rats were deeply anesthetized using sodium pentobarbital (60 mg/kg, i.p.; Euthatal; Merial Animal Health), then transcardially perfused with 0.1 M phosphate-buffered saline (PBS) followed by 4% paraformaldehyde in 0.1 M PBS (PFA). The brains were removed and placed in PFA for 4 h before being transferred to 25% sucrose and left overnight at room temperature, with gentle agitation. Four adjacent series of coronal sections (40 µm) were cut on a freezing sliding microtome. One series was mounted directly onto gelatin-coated slides after slicing, and was stained using cresyl violet, a Nissl stain, for verification of the specific brain regions.

A second series was used to stain the sections for NeuN. As this protein stains selectively for neurons (Mullen et al. 1992) it can sometimes clarify the extent of a lesion. Free-floating sections were rinsed in 0.1 M PBST (PBS with 0.2% Triton X-100) and treated with 0.3% H_2_O_2_ (hydrogen peroxide) in 0.1 M PBST for 10 min to suppress endogenous peroxidase activity. Sections were rinsed four times in 0.1 M PBST for 10 min each time, then incubated for 48 h at 4°C in the monoclonal anti-NeuN serum (1:5000; Chemican) diluted in PBST. After rinsing four times in 0.1 M PBST for a further 10 min each time, sections were incubated for 2 h at room temperature in biotinylated anti-mouse IgG (Vector Laboratories) and normal horse serum (Vector Laboratories) in PBST. Following four further 10-min washes in PBST, sections were transferred to avidin-biotin-horseradish peroxidase complex (1:200; ABC-Elite, Vector Laboratories) in PBST for 1 h. After four rinses in 0.1 M PBST and two rinses in 0.05 M Tris buffer, sections were left for 1–2 min in a chromagen solution consisting of 0.05% diaminobenzidine (Sigma), buffer solution, and 0.01% H_2_O_2_ (DAB substrate kit; Vector Laboratories). The reaction was monitored visually and stopped by rinsing in cold 0.1 M PBS. The sections were mounted and dried on gelatin-coated slides. All slides (Nissl and NeuN) were then dehydrated through an alcohol series, cleared with xylene, and cover-slipped using the mounting medium DPX.

#### Statistical methods

Statistical tests were carried out using SPSS 16.0 (SPSS Inc.). Where the assumption of sphericity was not met for parametric analysis, Greenhouse-Geisser corrections have been applied. The criterion α level was *P* ≤ 0.05.

### Experiment 1—cross-modal object recognition

#### Procedure

##### Apparatus

The Y-maze was constructed of wood and painted white. Each arm was 27 cm long and 10 cm high; the walls were 40 cm high. In the start arm there was a wooden sliding door set 18 cm from the end of the arm which, when closed, created an area within which an animal could be held until the beginning of a trial. There were also two clear Perspex sliding doors, positioned 9 cm from the end of each sample arm that could be lowered during trials in the light to prevent the animal from using tactile cues to explore the objects (see Fig. 1). These same barriers were removed during trials in the dark. During light trials, room illumination was provided by overhead fluorescent lights. During dark trials, room illumination was provided by infrared spotlights, with the experimenter using night-vision goggles (Productive Firm Dipol Ltd.) in order to see. Mean light intensity in the center of the maze during light trials was 590 l×, and during dark trials was less than 1 l×. A video camera sensitive to infrared light was mounted on the ceiling to record each trial. This camera, which was connected to a monitor/DVD recorder, recorded the rats’ behavior.

For the recognition tests, three identical copies of objects made from plastic, glass, aluminum, or ceramic were used. These objects varied in height from 10 to 40 cm, and differed in their tactile and visual properties. Before any object was placed in the maze it was wiped down with 50% ethanol to limit odor cues.

##### Pre-training

The cross-modal object recognition task started 4 mo after surgery for the RSdysg and Sham1 cohort. Prior to this experiment, the rats had been tested on spatial tasks in a radial-arm maze and water maze, as well as an object-spatial recognition problem in the bowtie maze (Albasser et al. 2010). None of the objects used for that final task was similar to an object used in the experiments reported here. For the RScomp and Sham2 cohort, cross-modal object recognition testing began 3 mo after surgery. The animals had previously been tested on a go/no-go task involving spatial discrimination.

Habituation to the Y-maze took place over two consecutive days. No objects were present in the maze during habituation. On each day the rats were brought to the testing room from the holding room in an individual carrying box made of metal, with a lid that prevented the animal from seeing the room. Each rat spent two sessions in the maze consisting of 5 min in the dark with the Perspex barriers removed and 5 min in the light with the barriers in place. The rats were removed from the maze between each session and the order in which the sessions took place was counterbalanced across days and by lesion group.

##### Recognition testing

The experiment consisted of either four different trial types (Cohort 1) or three different trial types (Cohort 2), each of which was repeated twice. Each test trial consisted of two phases, a sample phase and a choice phase. The sample phase was 3 min long and the choice phase lasted 1 min. The greatest preference for the novel object is usually seen during the first minute, diminishing after that time (Dix and Aggleton 1999), so this duration was chosen to give the clearest preference. The sample and choice phases were separated by a retention interval of 15 min for the RSdysg and Sham1 groups, and by 5 min for the RScomb and Sham2 groups. The rats in Cohort 2 were given a shorter retention interval to help counter potential floor effects. For Cohort 1, there were four different classes of recognition test, reflecting the fact that both the sample phase and test phase could be in the light or the dark. The four trial types were designated: light–light, light–dark, light–dark, dark–dark, where the first word refers to the sample condition and the second word refers to the test condition. It was found that the Cohort 1 rats could not recognize objects in the light–dark condition, so this trial type was not given to Cohort 2.

At the beginning of the sample phase the rat was placed in the start area of the Y-maze and the sliding door was raised. The trial began when the whole of the rat's body, excluding the tail, left the start area. The sliding door was then closed and the rat was allowed to explore the objects. During the sample phases, identical objects were placed at the end of each arm. After 3 min the rat was removed from the maze and returned to the carrying box for the duration of the retention interval.

During the retention interval, one of the objects in the Y-maze was replaced with an identical copy and the other replaced with a novel object that the rat had not yet explored. The arm in which the novel object was placed was counterbalanced across animals and across trials. To begin the choice phase the rat was placed back in the start area of the Y-maze and the sliding door opened. Once the rat had left the start area it was given 1 min to explore the two different objects. Rats were tested twice on each of the various trial types (light–light, dark–dark, dark–light, and light–dark), with a minimum of 48 h between tests (see Fig. 1). Different objects were used for each of the rat's two trials.

Videos of the rats’ behavior during the sample and choice phases were used to score how long rats spent exploring each object. In the dark, exploration was defined as time spent with the nose pointing toward the object at a distance of <1 cm; in the light exploration was defined as time spent with the nose pointing to the area of the Perspex barrier directly in front of the object. Behavioral scoring was carried out with the experimenter blind to the lesion status of the animal.

### Experiment 2—visual object recognition

#### Procedure

##### Object screening (dark–dark testing)

The goal of the Experiment was to use pairs of different objects that could only be distinguished by their visual appearance. For this reason, all pairs of objects were expected to be indistinguishable by shape, texture, and smell. Examples included a Coke can versus a Diet Coke can, or two glass containers of different flavors of the fruit drink, Schloer (Merrydown) (see Fig. 6 for examples). These objects should, therefore, be identical aside from their visual features. A second, related criterion was that these objects should not be discriminable in the dark and any such examples were excluded (see below). The first stage of the experiment, therefore, consisted of a series of screening trials run in the dark, using the same dark–dark protocol described for Experiment 1. The D2 scores for each pair of objects were calculated following testing, and any objects that the rats could recognize in the dark were removed from the experiment as they could be distinguished non-visually. Object pairs that could not be distinguished in the dark were subsequently used for testing in the light.

Experiment 2 took place immediately after Experiment 1 using the same test room and Y-maze apparatus, so no additional habituation was required. On each test day the rats were brought to the testing room from the holding room in an individual carrying box as previously described. During the sample phase, identical objects were placed at the end of each arm of the Y-maze. After 3 min the rat was removed from the maze and returned to the holding box for 5 min. During this time, one of the objects was replaced with an identical copy and the other with a novel object to which the rat had not yet been exposed. The arm in which the novel object was placed was counterbalanced across animals and across trials. At the end of 5 min the rat was then returned to the maze, which now contained two objects (one familiar) that were visually different but very alike in regard to their other sensory properties. The rat was allowed to explore these objects and was removed from the maze after 1 min. The mean light intensity in the center of the maze during dark trials was <1 lx.

Rats were tested with five different sets of suitable objects, with an interval of at least 48 h between sessions. Of these five, two sets of objects could be distinguished in the dark and so were not tested in the light.

##### Visual recognition (light–light testing)

This condition used sets of objects that could not be distinguished in the dark (see above). At least 48 h were left between testing on each set of objects. The procedure was identical to that used during prescreening, except that the room was illuminated. The light intensity in the apparatus was 590 l×.

## References

[HINDLEYLM032516C1] Aggleton JP 2010 Understanding retrosplenial amnesia: Insights from animal studies. Neuropsychologia 48: 2328–2338 19800900 10.1016/j.neuropsychologia.2009.09.030

[HINDLEYLM032516C2] Albasser MM, Chapman RJ, Amin E, Iordanova MD, Vann SD, Aggleton JP 2010 New behavioral protocols to extend our knowledge of rodent object recognition memory. Learn Mem 17: 407–419 20682810 10.1101/lm.1879610 PMC2920753

[HINDLEYLM032516C3] Albasser MM, Amin E, Iordanova MD, Brown MW, Pearce JM, Aggleton JP 2011 Separate but interacting recognition memory systems for different senses: The role of the rat perirhinal cortex. Learn Mem 18: 435–443 21685150 10.1101/lm.2132911 PMC3125609

[HINDLEYLM032516C4] Albasser MM, Olarte-Sánchez C, Amin E, Horne MR, Newton MJ, Warburton EC, Aggleton JP 2013 The neural basis of nonvisual object recognition memory in the rat. Behav Neurosci 127: 70–85 23244291 10.1037/a0031216 PMC3569044

[HINDLEYLM032516C5] Banati RB, Goerres GW, Tjoa C, Aggleton JP, Grasby P 2000 The functional anatomy of visual-tactile integration in man: A study using positron emission tomography. Neuropsychologia 38: 115–124 10660224 10.1016/s0028-3932(99)00074-3

[HINDLEYLM032516C6] Burgess N, Becker S, King JA, O'Keefe J 2001 Memory for events and their spatial context: Models and experiments. Philos Trans R Soc Lond B Biol Sci 356: 1493–1503 11571039 10.1098/rstb.2001.0948 PMC1088531

[HINDLEYLM032516C7] Burn CC 2008 What is it like to be a rat? Rat sensory perception and its implications for experimental design and rat welfare. Appl Anim Behav Sci 112: 1–32

[HINDLEYLM032516C8] Byrne P, Becker S, Burgess N 2007 Remembering the past and imagining the future: A neural model of spatial memory and imagery. Psychol Rev 114: 340–375 17500630 10.1037/0033-295X.114.2.340 PMC2678675

[HINDLEYLM032516C9] Chen LL, Lin LH, Green EJ, Barnes CA, McNaughton BL 1994 Head-direction cells in the rat posterior cortex. I. Anatomical distribution and behavioral modulation. Exp Brain Res 101: 8–23 7843305 10.1007/BF00243212

[HINDLEYLM032516C10] Cooper BG, Mizumori SJ 1999 Retrosplenial cortex inactivation selectively impairs navigation in darkness. Neuroreport 10: 625–630 10208601 10.1097/00001756-199902250-00033

[HINDLEYLM032516C11] Deacon TW, Eichenbaum H, Rosenberg P, Eckmann KW 1983 Afferent connections of the perirhinal cortex in the rat. J Comp Neurol 220: 168–190 6643724 10.1002/cne.902200205

[HINDLEYLM032516C12] Deegan JF, Jacobs GH 1993 On the identity of the cone types of the rat retina. Exp Eye Res 56: 375–377 8472794 10.1006/exer.1993.1049

[HINDLEYLM032516C13] Dix SL, Aggleton JP 1999 Extending the spontaneous preference test of recognition: Evidence of object-location and object-context recognition. Behav Brain Res 99: 191–200 10512585 10.1016/s0166-4328(98)00079-5

[HINDLEYLM032516C14] Ennaceur A, Delacour J 1988 A new one-trial test for neurobiological studies of memory in rats. 1: Behavioral data. Behav Brain Res 31: 47–59 3228475 10.1016/0166-4328(88)90157-x

[HINDLEYLM032516C15] Ennaceur A, Neave N, Aggleton JP 1997 Spontaneous object recognition and object location memory in rats: The effects of lesions in the cingulate cortices, the medial prefrontal cortex, the cingulum bundle and the fornix. Exp Brain Res 113: 509–519 9108217 10.1007/pl00005603

[HINDLEYLM032516C16] Gonzalez CLR, Whishaw IQ, Kolb B 2003 Complete sparing of spatial learning following posterior and posterior plus anterior cingulate cortex lesions at 10 days of age in the rat. Neuroscience 122: 563–571 14614920 10.1016/s0306-4522(03)00295-1

[HINDLEYLM032516C17] Goulet S, Murray EA 2001 Neural substrates of crossmodal association memory in monkeys: The amygdala versus the anterior rhinal cortex. Behav Neurosci 115: 271 11345954

[HINDLEYLM032516C18] Holdstock JS, Hocking J, Notley P, Devlin JT, Price CJ 2009 Integrating visual and tactile information in the perirhinal cortex. Cereb Cortex 19: 2993–3000 19386635 10.1093/cercor/bhp073 PMC2774401

[HINDLEYLM032516C19] Insausti R, Herrero MT, Witter MP 1997 Entorhinal cortex of the rat: Cytoarchitectonic subdivisions and the origin and distribution of cortical efferents. Hippocampus 7: 146–183 9136047 10.1002/(SICI)1098-1063(1997)7:2<146::AID-HIPO4>3.0.CO;2-L

[HINDLEYLM032516C20] Keene CS, Bucci DJ 2008 Neurotoxic lesions of retrosplenial cortex disrupt signaled and unsignaled contextual fear conditioning. Behav Neurosci 122: 1070–1077 18823164 10.1037/a0012895

[HINDLEYLM032516C21] Kobayashi Y, Amaral DG 2003 Macaque monkey retrosplenial cortex: II. Cortical afferents. J Comp Neurol 466: 48–79 14515240 10.1002/cne.10883

[HINDLEYLM032516C22] Kobayashi Y, Amaral DG 2007 Macaque monkey retrosplenial cortex: III. Cortical efferents. J Comp Neurol 502: 810–833 17436282 10.1002/cne.21346

[HINDLEYLM032516C24] Mullen RJ, Buck CR, Smith AM 1992 NeuN, a neuronal specific nuclear protein in vertebrates. Development 116: 201–211 1483388 10.1242/dev.116.1.201

[HINDLEYLM032516C25] Neave N, Lloyd S, Sahgal A, Aggleton JP 1994 Lack of effect of lesions in the anterior cingulate cortex and retrosplenial cortex on certain tests of spatial memory in the rat. Behav Brain Res 65: 89–101 7880459 10.1016/0166-4328(94)90077-9

[HINDLEYLM032516C26] Parron C, Save E 2004 Comparison of the effects of entorhinal and retrosplenial cortical lesions on habituation, reaction to spatial and non-spatial changes during object exploration in the rat. Neurobiol Learn Mem 82: 1–11 15183166 10.1016/j.nlm.2004.03.004

[HINDLEYLM032516C27] Paxinos G, Watson C 2007 The rat brain in stereotaxic coordinates. Elsevier Academic Press, San Diego, CA

[HINDLEYLM032516C28] Pothuizen HHJ, Aggleton JP, Vann SD 2008 Do rats with retrosplenial cortex lesions lack direction? Eur J Neurosci 28: 2486–2498 19032585 10.1111/j.1460-9568.2008.06550.x

[HINDLEYLM032516C29] Pothuizen HHJ, Davies M, Albasser MM, Aggleton JP, Vann SD 2009 Granular and dysgranular retrosplenial cortices provide qualitatively different contributions to spatial working memory: Evidence from immediate-early gene imaging in rats. Eur J Neurosci 30: 877–888 19712100 10.1111/j.1460-9568.2009.06881.x

[HINDLEYLM032516C30] Reid MJ, Jacklin DL, Winters BD 2012 Crossmodal object recognition in rats with and without multimodal object pre-exposure: No effect of hippocampal lesions. Neurobiol Learn Mem 98: 311–319 22975081 10.1016/j.nlm.2012.09.001

[HINDLEYLM032516C31] Reid JM, Jacklin DL, Winters BD 2013 Delineating prefrontal cortex region contributions to crossmodal object recognition in Rats. Cereb Cortex 10.1093/cercor/bht061 23505287

[HINDLEYLM032516C32] Robinson S, Keene CS, Iaccarino HF, Duan D, Bucci DJ 2011 Involvement of retrosplenial cortex in forming associations between multiple sensory stimuli. Behav Neurosci 125: 578–587 21688884 10.1037/a0024262 PMC3144268

[HINDLEYLM032516C33] Van Groen T, Wyss JM 1990 Connections of the retrosplenial granular a cortex in the rat. J Comp Neurol 300: 593–606 2273095 10.1002/cne.903000412

[HINDLEYLM032516C34] Van Groen T, Wyss JM 1992 Connections of the retrosplenial dysgranular cortex in the rat. J Comp Neurol 315: 200–216 1545009 10.1002/cne.903150207

[HINDLEYLM032516C35] Van Groen T, Wyss JM 2003 Connections of the retrosplenial granular b cortex in the rat. J Comp Neurol 463: 249–263 12820159 10.1002/cne.10757

[HINDLEYLM032516C36] Vann SD, Aggleton JP 2002 Extensive cytotoxic lesions of the rat retrosplenial cortex reveal consistent deficits on tasks that tax allocentric spatial memory. Behav Neurosci 116: 85–94 11895186

[HINDLEYLM032516C37] Vann SD, Aggleton JP 2004 Testing the importance of the retrosplenial guidance system: Effects of different sized retrosplenial cortex lesions on heading direction and spatial working memory. Behav Brain Res 155: 97–108 15325783 10.1016/j.bbr.2004.04.005

[HINDLEYLM032516C38] Vann SD, Aggleton JP 2005 Selective dysgranular retrosplenial cortex lesions in rats disrupt allocentric performance of the radial-arm maze task. Behav Neurosci 119: 1682–1686 16420172 10.1037/0735-7044.119.6.1682

[HINDLEYLM032516C39] Vann SD, Wilton LAK, Muir JL, Aggleton JP 2003 Testing the importance of the caudal retrosplenial cortex for spatial memory in rats. Behav Brain Res 140: 107–118 12644284 10.1016/s0166-4328(02)00274-7

[HINDLEYLM032516C40] Vann SD, Aggleton JP, Maguire EA 2009 What does the retrosplenial cortex do? Nat Rev Neurosci 10: 792–802 19812579 10.1038/nrn2733

[HINDLEYLM032516C41] Vargha-Khadem F, Gadian DG, Watkins KE, Connelly A, Van Paesschen W, Mishkin M 1997 Differential effects of early hippocampal pathology on episodic and semantic memory. Science 277: 1117 9219696 10.1126/science.277.5324.376

[HINDLEYLM032516C42] Vogt BA, Miller MW 1983 Cortical connections between rat cingulate cortex and visual, motor, and postsubicular cortices. J Comp Neurol 216: 192–210 6863602 10.1002/cne.902160207

[HINDLEYLM032516C43] Whishaw IQ, Maaswinkel H, Gonzalez CLR, Kolb B 2001 Deficits in allothetic and idiothetic spatial behavior in rats with posterior cingulate cortex lesions. Behav Brain Res 118: 67–76 11163635 10.1016/s0166-4328(00)00312-0

[HINDLEYLM032516C44] Winters BD, Reid JM 2010 A distributed cortical representation underlies crossmodal object recognition in rats. J Neurosci 30: 6253–6261 20445051 10.1523/JNEUROSCI.6073-09.2010 PMC6632708

[HINDLEYLM032516C45] Wyss JM, Van Groen T 1992 Connections between the retrosplenial cortex and the hippocampal formation in the rat: A review. Hippocampus 2: 1–11 1308170 10.1002/hipo.450020102

